# A meta-review of methods of measuring and monitoring safety in primary care

**DOI:** 10.1093/intqhc/mzab117

**Published:** 2021-08-18

**Authors:** Paul O’Connor, Caoimhe Madden, Emily O’Dowd, Dara Byrne, SinÉad Lydon

**Affiliations:** Discipline of General Practice, School of Medicine, National University of Ireland Galway, Galway H91 TK33, Ireland; Irish Centre for Applied Patient Safety and Simulation, National University of Ireland Galway, Co Galway H91 TK33, Ireland; Discipline of General Practice, School of Medicine, National University of Ireland Galway, Galway H91 TK33, Ireland; Irish Centre for Applied Patient Safety and Simulation, National University of Ireland Galway, Co Galway H91 TK33, Ireland; Discipline of General Practice, School of Medicine, National University of Ireland Galway, Galway H91 TK33, Ireland; Irish Centre for Applied Patient Safety and Simulation, National University of Ireland Galway, Co Galway H91 TK33, Ireland; Irish Centre for Applied Patient Safety and Simulation, National University of Ireland Galway, Co Galway H91 TK33, Ireland; School of Medicine, National University of Ireland Galway, Co Galway H91 TK33, Ireland; Discipline of General Practice, School of Medicine, National University of Ireland Galway, Galway H91 TK33, Ireland; Irish Centre for Applied Patient Safety and Simulation, National University of Ireland Galway, Co Galway H91 TK33, Ireland

**Keywords:** patient safety, primary care, measurement, monitoring, meta-review

## Abstract

**Background:**

A major barrier to safety improvement in primary care is a lack of safety data. The aims of this systematic meta-review (registration: CRD42021224367) were to identify systematic reviews of studies that examine methods of measuring and monitoring safety in primary care; classify the methods of measuring and monitoring safety in the included systematic reviews using the five safety domains of Vincent *et al.*’s framework and use this information to make recommendations for improving the measurement and monitoring of safety in primary care.

**Methods:**

Four databases (Medline, Academic Search Complete, Web of Science and CINAHL) and the grey literature were screened in November 2020, with searches updated in January 2021. Systematic reviews were included if they addressed the measurement of patient safety in primary care and were published in English. Studies were assessed using the Critical Appraisal Skills Programme for systematic reviews.

**Results:**

A total of 6904 papers were screened, with 13 systematic reviews included. A commonly reported method of measuring ‘past harm’ was through patient record review. The most frequent methods for assessing the ‘reliability of safety critical processes’ were checklists, observations and surveys of staff. Methods used to assess ‘sensitivity to operations’ included observation, staff surveys, interviews, focus groups, active monitoring and simulated patients. Safety climate surveys were a commonly used as an approach to assess ‘anticipation and preparedness’. A number of the reviews concluded that safety data could, and should, be used for ‘integration and learning’. The main limitation of the meta-review was that it was of systematic reviews only.

**Conclusions:**

Many of the methods for measuring and monitoring safety are readily available, quick to administer, do not require external involvement and are inexpensive. However, there is still a need to improve the psychometric properties of many measures. Researchers must support the development of psychometrically sound safety measures that do not over burden primary care practitioners. Policymakers must consider how primary care practitioners can be supported to implement these measures.

## Introduction

Improving patient safety and reducing preventable harm in healthcare is an ongoing challenge. A barrier to safety improvement is the lack of data to allow organizations, teams and individual healthcare providers to evaluate how they are performing and where there are deficits and risks [1]. Recognizing the challenges of measuring and monitoring safety (MMS), Vincent *et al.* developed the MMS framework (see Table 1) [2, 3]. The MMS framework provides a useful approach to considering methods of MMS in a particular domain of healthcare and identifying where there may be deficiencies in, or opportunities for, MMS [4].

**Table 1:** Description of the five dimensions of safety (adapted from Vincent *et al.* [2])

MSS dimension	Purpose	Examples
‘Past harm’ Has patient care been safe in the past?	Assess rates of past harm to patients	Patient record review Adverse event reports
‘Reliability’ Are clinical systems and processes reliable?	Assess the reliability of safety critical processes and the ability of staff to follow these procedures	Observations of safety critical behaviour Practice safety checklist
‘Sensitivity to operations’ Is care safe today?	Support the monitoring of safety on an hourly or daily basis	Observations and conversations with practice staff Talking to patients
‘Anticipation and preparedness’ Will care be safe in the future?	Support the anticipation and respond to future threats to safety	Safety climate assessment Structured reflection
‘Integrating and learning’ Are we responding and improving?	Analyse and use safety information to improve safety	Aggregate data on patient complaints Feedback and implementation of safety lessons

Research on patient safety, and MMS, in primary care has lagged behind that of secondary care. This is arguably due to the perception that primary care is relatively low risk. However, it has been found that 2–3% of primary care consultations result in a patient safety incident (PSI), with ∼4% of these PSIs associated with severe harm [5]. Given that ∼90% of all healthcare contacts occur in primary care, there is a large potential for patient harm to occur [6]. Primary care providers have stated that they do not know how to improve safety [7]. However, it has been found that when primary care providers are provided with data identifying the safety issue in their practice, they are able to identify and implement methods to effectively address these deficits [8, 9]. Therefore, there is a need to identify valid, reliable, readily available and easily administered methods of MMS. We believe that an effective way to do this is through a meta-review of systematic reviews of methods of MMS in primary care.

A meta-review summarizes the evidence from multiple research syntheses, compares the findings from the systematic reviews and assesses whether the review authors reached similar or contradictory conclusions [10]. The purpose of a meta-review is not to repeat what was done in the previous systematic reviews (e.g. duplicate the searches). Rather, it is to provide an overview of the research evidence on a particular issue [10]. Therefore, the aims of our systematic meta-review were to

1. identify systematic reviews of studies of MMS in primary care
2. use the five dimensions of safety from Vincent *et al.*’s [2, 3] framework to classify the methods of MMS in primary care reported in the systematic reviews and
3. based on the classification of the methods of MMS and the conclusions of the authors of the reviews make recommendations for MMS in primary care.

## Method

The meta-review was prospectively registered with the International Prospective Register of Systematic Reviews (registration number: CRD42021224367). This review is reported in accordance with the Preferred Reporting Items for Systematic Reviews and Meta-Analyses (PRISMA) guidelines [11].

### Design

This study was a meta-review of systematic reviews of patient safety measures in primary care.

### Inclusion and exclusion criteria

Systematic reviews were included if they focused on studies about MMS in primary care and were published in English. Studies were excluded if they were another form of study such as a narrative review or original research, not published in English, focused on patient safety interventions or any aspects of patient safety other than measurement or monitoring or it was not possible to access the primary care-specific data within a larger synthesis.

### Search strategy

A search strategy was developed with the support of a research librarian and adapted for the different databases (see Supplemental Material 1 for the Medline search strategy). Grey literature was also searched via Google Scholar, with the terms ‘Primary care, measure, patient safety, systematic review’ and ‘general practice, patient safety, measure, and systematic review’.

### Procedure

Four databases (Medline, Academic Search Complete, Web of Science and CINAHL) were screened in November 2020, and searches were re-run in January 2021. The databases were searched by two authors (CM and EOD). Google Scholar was searched by one author (EOD). Titles and abstracts were screened, and full texts were examined of papers about which the authors had queries or which appeared suitable for inclusion. All disagreements on inclusion/exclusion were resolved with the input of all authors. Data were extracted from the systematic reviews by two authors independently (CM and EOD) and agreed upon. The data extracted were as follows: authors, year, aim, number of included articles, search range, language, database, methods of MMS, quality appraisal findings and a summary of the results. The methods of MMS summarized in the systematic reviews were classified using Vincent *et al.*’s [2, 3] dimensions of MMS by agreement between two authors (POC and SL).

### Critical appraisal

The Critical Appraisal Skills Programme (CASP) systematic review checklist [12] was used to assess the quality of the included systematic reviews. The rationale for using the CASP systematic review checklist was that it included clear and explicit guidance for what to consider when responding to the items in the CASP checklist for each study and could be applied to the types of systematic reviews (i.e. reviews focused on measurement rather than interventions) included in our meta-review with minimal adaptation required to the CASP checklist. The CASP systematic review checklist has three parts: part A, are the results of the study valid? (five items); part B, what are the results? (two items); and part C, will the results help locally (in the case of this review interpreted as improving safety in primary care; three items). One item from part B and one from part C were not applied in our appraisal as they are relevant for interventions. For each item a response of ‘yes’ (1), ‘no’ (0) or ‘can’t tell’ (0) was given. Therefore, each systematic review was given a score out of 8. The systematic reviews were independently appraised by two reviewers (POC and EOD), with any disagreements resolved via discussion.

## Results

**Figure 1:**
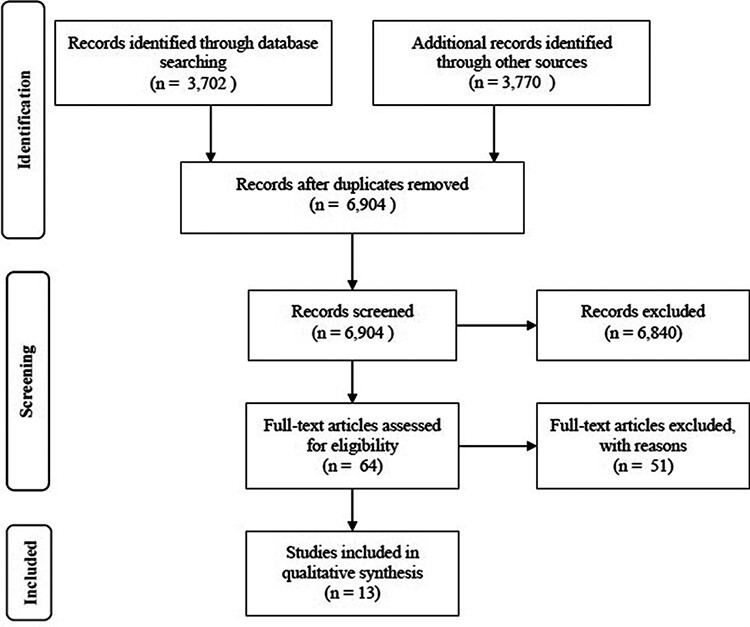
PRISMA flow diagram.

A total of 6904 papers were screened, with 13 systematic reviews included. Examples of systematic reviews that were not included are as follows: Manser *et al*. [13] (unable to extract primary care specific data) and Verbekel *et al*. [14] (focused on interventions and not methods of MMS). The PRISMA flow diagram of included studies can be found in Figure 1. Tables 2 and 3 provide a summary of these included reviews, with a more detailed description provided in Supplemental Material 2.

**Table 2:** Systematic review characteristics

	*N*
Number of reviews	13
All safety measures	4
Safety climate measures only	4
Reporting systems only	2
Patient record review only	3
Number of studies included in the reviews	
Mean	24.7
SD	12.0
Range	14–56
Number of databases searched	
Mean	4.4
SD	1.9
Range	1–8

**Table 3:** Summary of included systematic reviews

Authors	Year	# articles	Aim of review	Summary of findings
All safety measures				
Hatoun *et al.* [18]	2017	21	To identify published articles detailing safety measures applicable to adult primary care	– Although numerous measures of patient safety exist, many are not validated and pertain only to a particular research study or quality improvement project
Lawati *et al.* [19]	2018	28	To review the literature on the safety culture and patient safety measures used globally	– The most common theme emerging from 2011 onwards was the assessment of safety culture – The most commonly used safety culture assessment tool was the Hospital Survey on Patient Safety Culture
Lydon *et al.* [15]	2017	56	To identify and review articles that presented or described the use of measures of patient safety suitable for use in general practice settings	– There is a need to improve the psychometric properties of existing tools as opposed to developing new tools – There is a need to take a multi-methods approach to assessing patient safety
Marchon and Mendes [16]	2014	33	To identify methodologies to evaluate incidents in primary health care, types of incidents, contributing factors and solutions to make primary care safer	– Highlighted the need for expanding safety culture in primary care in order to prepare patients and health professionals to identify and manage adverse events
Safety climate measures only				
Curran *et al.* [24]	2018	17	To identify the origins, psychometric properties, quality and safety climate domains measured by survey instruments used to assess safety climate in primary care settings	– Consideration should be given to selecting an instrument that has safety climate domains relevant to primary care – Need to focus on further establishing the criterion-related validity of existing surveys, rather than creating new surveys – Questionnaire with the most evidence of validity and reliability: PC SafeQuest, Frankfurt Patient Safety Climate Questionnaire and SCOPE
Desmedt *et al.* [26]	2018	14	To give an overview of empirical studies using self-reported instruments to assess patient safety culture in primary care and to synthesize psychometric properties of these instruments	– A standard and widely validated survey is needed to increase generalizability and comparability – The SCOPE-PC survey is the most appropriate instrument to assess patient safety culture in primary care – There is a need to consider the triangulation of qualitative and quantitative methods to attain an in-depth assessment of culture
Madden *et al.* [27]	2020	44 (10)^a^	To identify patient-reported safety climate measures described in the literature and make recommendations for best practice	– Few measures reported satisfactory levels of validity, reliability or usability measurement – Few measures are specifically designed for measuring the attitudes of primary care patients – There is value in using a mixed-methods approach to measuring patient safety
Vasconcelos *et al.* [25]	2018	18	To conduct an investigation of the tools used to assess safety culture in primary care	– In addition to reliability, other measures of validity are needed to establish the credibility of an instrument. Research addressing other types of psychometric tests is needed – The domains of communication, management perception and teamwork were present in all instruments. Future research on patient safety should incorporate these attributes
Reporting systems only				
King *et al.* [22]	2010	17 (5)^a^	To identify the state of the art in patient reporting systems used in research studies	– When designing a reporting tool, it should be evaluated in the local setting to ensure appropriate terminology is used. International terminology standards should be adopted. Reports from patients should be actively solicited
Ricci-Cabello *et al.* [23]	2015	28	To identify and characterize available patient-reported instruments to measure patient safety in primary care	– Taxonomies for classifying errors and harm were not consistently used for developing the instruments, impairing the ability to make comparisons – There was a lack of valid and reliable instruments specifically designed to provide a comprehensive measurement of the safety of care provided in primary care practices
Patient record review only				
Davis *et al.* [17]	2018	15	To understand the ability of trigger tools to detect preventable adverse events in the primary care outpatient setting	– Outcome measures were heterogenous, precluding the ability to quantitatively compare the studies
Madden *et al.* [20]	2018	15	This review aimed to synthesize the literature describing the use of patient record review to measure and improve patient safety in primary care	– Studies using trigger tool methodologies tended to detect higher incidences of PSIs, suggesting greater empirical support than other methods – Need to refine and standardize the methods used in patient record review to improve consistency and validity and facilitate ease of comparison across studies – Strong rationale for combining more than one method of studying patient safety
Tsang *et al.* [21]	2012	15	To determine the types of adverse events that are routinely recorded in primary care	– Measurement of primary care adverse events was often based on secondary care data in conjunction with other clinical and non-clinical information. This use of multiple data sources will enhance the accuracy of measurements and compensate weaknesses inherent to individual data types – Greater attention required on developing indicators and other measures that take advantage of the available IT resources to improve quality and safety

a Number in brackets is the number of included studies focused on primary care.

### Past harm

The most commonly reported method of measuring past harm was through patient record review. Papers using patient record review methodologies were included in seven reviews [15–31], with three reviews specifically focused on studies using patient record review methodologies [17, 20, 21]. Issues identified with the use of patient record review methodology were the potential for false positives, lack of tools and poor quality of studies [17, 20]. It was suggested that a trigger tool approach to record review may offer a reliable approach to reviewing patient records [17, 20]. However, there is a need to refine and standardize the methods used to improve consistency and validity and facilitate ease of comparison across studies [20].

Papers describing incident reporting systems or adverse event reports were included in four reviews [2, 16, 19, 22], with two of these reviews specifically focused on reports generated by patients [22, 23]. It was recognized that there is a need for further development and refinements of patient reporting systems [22, 23].

Other examples of methods of assessing past harm from papers included in the systematic reviews were as follows: the use of pharmacy and administrative data (described in one review [16]); interviews, surveys and focus groups with patients about their experiences of harm (included in two reviews [13, 14]) and staff surveys or interviews (included in one review [16]).

### Reliability of safety clinical processes

Papers describing methods of measuring and monitoring the reliability of safety critical processes were included in four systematic reviews [15, 16, 18, 19]. The most commonly reported methods were checklists, observations and surveys of staff (included in four reviews [15, 16, 18, 19]). Studies utilizing patient surveys (e.g. assessing for medication discrepancies) were included in two reviews [18, 19]. It was suggested that checklists should be accompanied by structured guidelines for use that will increase the ease of employment, allowing them to be implemented at a relatively low cost [15].

### Sensitivity to operations

Two systematic reviews included papers that were concerned with sensitivity to operations [15, 16]. These reviews included papers that used observation, staff surveys, interviews or focus groups (included in [16]) and active monitoring in which primary care providers completed an assessment immediately after a consultation to identify any potential harm (included in [15]). Studies in which simulated patients presented specific cases to assess the care provided by the primary care provider were included in two reviews [15, 16]. It was suggested that the reason why active-monitoring and simulated patients were infrequently used is because of the resource intensive nature of these approaches [15].

### Anticipation and preparedness

Seven reviews included papers reporting the use of safety climate surveys [15, 16, 19, 24–27], with three reviews completely devoted to staff surveys [24–26] and one on patient assessments of safety climate [27]. There are a large number of different safety climate surveys that have been used in primary care setting (e.g. Curran *et al.* [24], included 17 different surveys in their review). A number of the reviews concluded that the psychometric properties of these surveys are variable [15, 25–27]. Particular staff safety climate survey with the most evidence of validity and reliability were the PC SafeQuest, Frankfurt Patient Safety Climate Questionnaire (FraSiK) and SCOPE. It was suggested that rather than developing new surveys, researchers should focus on improving the psychometric properties of existing tools [15, 24, 26].

Other less frequently used methods of assessing anticipation and preparedness included the following: staff surveys of patient safety (e.g. medical office survey on patient safety; included in one review [16]), staff interviews or focus groups (included in two reviews [16, 19]) and Failure Modes and Effects Analysis (included in one review [19]). It was suggested that the advantage of interview methodologies was that the interviewer’s proximity to the person they are interviewing allows an analysis of the impact of a direct or indirect event or experience [16]. However, issues such as geographical separation, sampling and resources were recognized as a barrier to interview approaches.

### Integration and learning

Two reviews included studies that addressed the integration and learning dimension of safety [16, 19]. One review [16] included a study concerned with identifying lessons learned from error, and another review included studies on the use of safety culture data to inform risk management and feedback in order to inform improvement efforts [19]. A number of the reviews also concluded that safety data should be used to inform patient safety improvement [16, 19], and there was a need to triangulate safety data from multiple sources [15, 20, 21, 26, 27]. However, it was suggested that the recruitment of patients to complete patient report measures may add another layer of difficulty that may reduce the use of such measures [15].

### Quality assessment

The mean CASP score was 7.3/8 (SD = 1.0; range = 5–8). CASP scores for individual studies are presented in Supplemental Material 2. The reviews generally addressed whether the results of the review were valid and were judged to have included the relevant papers. However, three of the reviews [16, 23, 25] could have had more specific research questions, and four of the reviews [18, 21, 23, 25] could have carried out a more rigorous quality assessment. All of the reviews clearly presented the findings of the review, the findings were relevant to safety improvement in primary care and all of the outcomes have been adequately considered.

## Discussion

### Statement of principal findings

A total of 13 systematic reviews of methods of MMS in primary care were included in this meta-review. Many of the methods for MMS in primary care are readily available, quick to administer, do not require external involvement and are inexpensive [15]. However, there is still a need to improve the psychometric properties of many of these methods for MMS [15, 18, 20, 23–26]. Therefore, with the exception of the sensitivity to operations dimension of safety, rather than developing new methods of MMS, there should instead be a focus on using and adapting existing methods of MMS in order to increase generalizability and comparability [15, 18, 24, 26]. There is also a need for multi-methods approach to measuring safety to assess safety across each of the five dimensions of safety described by Vincent *et al*. [2, 3].

### Strengths and limitations

The strengths of this meta-review are the broad coverage of methods of MMS in primary care, the prospective registration of the review protocol, the use of a comprehensive search strategy across multiple databases (including the grey literature) and a rigorous review process. The main limitation of the meta-review was that it was of systematic reviews only. Therefore, it does not include any methods of MMS that have not been included in a systematic review, nor is there a discussion of the specific measures described in individual papers. Rather, the focus is upon the conclusions drawn by the systematic review authors. This is consistent with the goal of a meta-review to provide an overview of the research evidence on a particular issue [10].

### Interpretation within the context of the wider literature

A trigger tool approach to patient record review may offer a reliable and usable approach to evaluating past harm in primary care [17, 20]. A trigger tool is a checklist of a selected number of clinical ‘triggers’ (e.g. frequency of consultation) that a reviewer seeks to identify when screening medical records [28]. If a ‘trigger’ is identified in the record, then the reviewer scrutinizes it in more detail to assess whether an undetected PSI had occurred [29]. It is possible to review up to 20 records in 2–3 h, with most patient records taking <5 minutes to review [9]. Moreover, patient record review data are useful in helping primary care providers to identify where safety improvements are required [8, 9]. Therefore, it is recommended that a trigger tool chart review methodology has great potential as a measure of past harm, and the application of this approach merits further refinement and investigation in primary care settings.

The included systematic reviews summarized a number of methods of assessing the reliability of critical processes. It is suggested that safety checklists may provide a practical method for identifying safety issues that can be readily completed by one member of the practice staff. For example, Bowie *et al.* [30] developed a 22-item checklist that addresses medicine management, housekeeping, information systems, registration checks, patient access and identification, and health and safety. It is recommended that checklists such as this could be used periodically by a practice manager to support the identification of workplace hazards that impact patient safety and quality of care.

Methods of measuring and monitoring sensitivity to operations are arguably less well developed than those designed to assess the other dimensions of safety. In secondary care, methods of measuring and monitoring sensitivity to operations include safety walk rounds, ward rounds, briefings and debriefings [2, 3]. However, these approaches are not applicable to primary care. The methods used in primary care were somewhat unstructured (e.g. focus groups), time consuming (e.g. primary care providers completed an assessment immediately after a consultation) or unlikely to be broadly acceptable (patients as ‘secret shoppers’). Therefore, it is recommended sensitivity to operations is a particular dimension of safety that would benefit from the development of structured approaches to assessment.

Safety climate surveys were the dominant approach for MMS in the anticipation and preparedness safety dimension. Safety climate is regarded as the measurable component of the underlying safety culture at a given point in time [31]. Safety culture refers to the values, attitudes, norms, beliefs, practices, policies and behaviours around safety in an organization [32]. Safety climate surveys provide a feasible method to assess the safety of primary care practices. It is recommended that to assess safety over time and to make (inter)national comparisons, it is important that a survey has sound psychometric properties [24]. It is further recommended that any staff-completed safety climate survey is carried out in parallel with one completed by patients who may have a different perspective on safety than practice staff [27].

A common conclusion among the systematic review authors was the need to integrate safety data from multiple sources in order to inform learning [15, 20, 21, 26, 27]. There are examples of such approaches in the literature. For example, the Scottish Patient Safety Programme in Primary Care includes a trigger tool chart review (past harm) and a safety climate survey (anticipation and preparedness) [33]. Madden *et al.*’s [8] feasibility study of this programme added feedback during practice meeting (integration and learning) and could be further extended with a safety checklist (reliability of safety critical processes). Therefore, it is recommended that researchers give consideration as to how data from measures across all of the other four dimensions of safety can be integrated in order to inform learning.

### Implications for policy, practice and research

Any effective safety surveillance system must consider methods of MMS that address each of the five dimensions of safety identified by Vincent *et al*. [2, 3]. It has been suggested that healthcare stakeholders could get the information they need with 25% of what is currently being spent on measurement [34]. Therefore, a healthcare safety surveillance system should be efficient and measure only what matters [34]. This is particularly true for primary care where there are generally not dedicated risk and safety personnel.

Lack of time has been identified by primary care providers as a barrier to implementing safety interventions [8]. Therefore, an approach to MMS that is considered too burdensome or lacks credibility will not be implemented [35]. There is also a need to consider how to encourage the implementation of a robust safety monitoring system in primary care practices. Implementation could be encouraged through allowing the MMS activities to be counted towards continuing medical education, allowing the safety data to be used for mandatory annual audits, or reductions in indemnity insurance for practices that have a robust safety management system in place. How to support and encourage practices to collect safety data is an important consideration for policymakers and researchers.

## Conclusions

This meta-review has provided an overview of approaches to MMS in primary care in order to identify considerations that need to be addressed in order to develop a safety monitoring system for primary care practices. Primary care doctors have highlighted that a lack of data is a barrier to improving patient safety. Therefore, researchers must support the development of psychometrically sound measures that do not overburden primary care practitioners. Policymakers must consider how primary care practitioners can be supported to implement these measures.

## Supplementary Material

mzab117_Supp

## Data Availability

All data are either presented in the article or included in the supplemental material.
