# Supplementary material for: A meta-review of methods of measuring and monitoring safety in primary care
Source: Int J Qual Health Care. 2021 Aug 18;33(3):mzab117. doi: 10.1093/intqhc/mzab117 (PMC8397923; doi:10.1093/intqhc/mzab117)
Supplement: mzab117_Supp [file mzab117_supp.zip › Supplemental material 2.docx]

**Supplemental Material 2: Full data extraction table**

| Authors | Year | Aim of review | # articles | Search range | Language | Databases | Measures included | Summary of quality appraisal findings | Summary of review findings | CASP |
| --- | --- | --- | --- | --- | --- | --- | --- | --- | --- | --- |
| **All safety measures** | | | | | | | | | | |
| Hatoun et al | 2017 | To identify published articles detailing safety measures applicable to adult primary care. | 21 | 1999-2017 | English | Medline | Past harm  -Patient record review  -Review of pharmacy and administrative data.  Reliability of processes  -Clinician survey (e.g. survey of incidents in which medications are improperly stored)  -Patient survey (e.g. survey of medication discrepancies)  Anticipation & preparedness  -Staff survey (e.g. survey on patient safety) | Recorded whether studies were validated or not. A total of 8 were validated using expert consensus, 2 using predictive validity, and 11 were not validated. | - Many clearly defined and easily measured safety measures exist for use in the adult primary care setting.  -Certain dimensions of primary care safety are under-represented.  - Although numerous measures of patient safety exist, many are not validated and pertain only to a particular research study or quality improvement project.  - Additional development of measures are needed to capture the nuanced and unique patient safety landscape of primary care. | Yes: 5 items  No: 3 items (research question not specific; <2 databases searched; insufficient quality assessment |
| Lawati et al | 2018 | To review the literature on the safety culture and patient safety measures used globally to inform the development of safety culture among health care workers in primary care with a particular focus on the Middle East. | 28 | 2000- 2014 | English | Medline, Embase, CINAHL,  Scopus. | Past harm  -Incident reporting  -Patient record review  Reliability of processes  -Practice checklist  -Staff survey  -Patient survey  Anticipation & preparedness  -Staff interviews about safety culture  -Staff safety culture/climate survey  -Failure modes and effects analysis  Integration and learning  -Use of safety culture data to inform risk management  -Feedback and assessment of quality and safety in order to inform improvement efforts. | Cross-sectional studies were evaluated using Strengthening the Reporting of Observational studies in Epidemiology (STROBE). All 20 studies examined using STROBE covered all items of the instrument.  Systematic reviews were evaluated by the AMSTAR. The AMSTAR score for the 3 included systematic reviews were: 7/11, 8/11, and 9/11.  Two studies were assessed with the Effective Public Health Practice Project (EPHPP). For one, it was concluded that this was a strong study which highlighted, limitations and implications, for the other it was concluded that the global  rating of this paper was moderate  Quality of one paper not assessed as Delphi study. | -The most common theme emerging from 2011 onwards was the assessment of safety culture.  -The most commonly used safety culture assessment tool was the Hospital Survey on Patient Safety Culture.  -Safety culture in primary care should be assessed on a regular basis to evaluate the effectiveness of safety in health institutions. | Yes: 8 items |
| Lydon et al | 2017 | To identify  and review articles that presented or described the use of measures  of patient safety suitable for use in general practice settings. | 56 | Until Feb 2016 | English | OVID Medline, Embase, CINAHL, PsychINFO | Past harm  -Patient record review  -Patient surveys and interviews  Reliability of processes  -Practice assessment checklist  Sensitivity to operations  - Active monitoring by physicians  -Simulated patients  Anticipation & preparedness  -Staff safety climate survey | A total of 81% of studies were quantitative, 11.% were qualitative, and 8.%) were mixed-methods.  Across all studies, the mean QATSDD score was 21.8 (SD= 6.5).  Examining the QATSDD for each measure, the mean (SD) was:   - Staff survey or interview- 23.7 (6.2) - Patient chart audit- 19.3 (5) - Practice assessment checklist- 19 (8.5) - Patient survey or interview- 18 (4) - Active monitoring system- 21 (0) - Simulated patients- 19.5 (10.6) | -Many of the measures are readily available, quick to administer, do not require external involvement, and are inexpensive.  -Patients' inputs were largely missing from the included studies.  -There is a need to improve the psychometric properties of existing tools as opposed to developing new tools.  -There is a need to take a multi-methods approach to assessing patient safety. | Yes: 8 items |

| Authors | Year | Aim of review | # articles | Search range | Language | Databases | Measures included | Summary of quality appraisal findings | Summary of review findings | CASP  score |
| --- | --- | --- | --- | --- | --- | --- | --- | --- | --- | --- |
| Marchon & Mendes | 2014 | To identify methodologies to evaluate incidents in primary health care, types of incidents, contributing factors, and solutions to make primary care safer. | 33 | 2007-Nov 2012 | Portuguese, English, Spanish | Pubmed, Embase, Scopus, LILACS, SciELO, and the thesis database of the general agency for support and evaluation of graduate education | Past harm  -Staff incident reporting.  -Patient incident reporting  -Patient record review  -Patient surveys, interviews, or focus groups  -Staff surveys or interviews  Reliability of processes  -Observation  - Staff surveys, interviews, or focus groups  Sensitivity to operations  - Staff interviews  -Observation  -Simulated patients  Anticipation & preparedness  -Staff safety climate survey  -Staff safety culture interviews or focus groups.  Integration and learning  -Lesson learned from error | Studies were evaluated using Strengthening the Reporting of Observational studies in Epidemiology (STROBE):   - 14 (42.4%) articles fully covered items in the STROBE instrument. - 4 papers did not address limitations. - 2 did not provide interpretation of findings. - 3 did not provide financing details. - 7 did not give context/justification of method. - 5 did not give detail on participants. - 1 did not give outcome/ - 1 did not provide other analyses of the results/ | -Reporting systems for adverse events were the most common data source in the studies.  -The current study highlighted the need for expanding safety culture in primary care in order to prepare patients and health professionals to identify and manage adverse events, while raising awareness concerning their shared capacity for change, thereby reducing errors in primary care and tensions between health professionals and the population. | Yes: 8 items |

| Authors | Year | Aim of review | # articles | Search range | Language | Databases | Measures included | Summary of quality appraisal findings | Summary of review findings | CASP |
| --- | --- | --- | --- | --- | --- | --- | --- | --- | --- | --- |
| **Safety climate measures only** | | | | | | | | | | |
| Curran et al | 2018 | To identify the origins, psychometric  properties, quality, and safety climate domains measured by survey instruments used to assess safety climate in primary care settings. | 17 | Until Feb 2016 | English | OVID Medline, Embase, CINAHL, PsychINFO | Anticipation & preparedness  -Staff safety climate surveys | There was large variability in the quantity and quality of psychometric assessment of the instruments. Of the included instruments:   - 65% described evidence of content validity. - 59% reported evidence of construct validity. - 53% demonstrated criterion-related validity - 77% assessed reliability (alphas ranging from 0.43 to 0.94 across subscales).   Four survey instruments (PC SafeQuest, FraSik, SCOPE, Norwegian SAQ-AV) had evidence of psychometric assessment across all 4 parameters. | -Safety climate surveys adapted from another healthcare setting or country, outside of that which it was developed, require a thorough assessment of the psychometric properties of the survey in the new environment.  -Consideration should be given to selecting an instrument that has safety climate domains relevant to primary care.  -Need to focus on further establishing the criterion-related validity of existing surveys- rather than creating new surveys.  -Questionnaire with the most evidence of validity and reliability were the PC SafeQuest. Frankfurt Patient Safety Climate Questionnaire (FraSiK), and SCOPE. | Yes: 8 items |

| Authors | Year | Aim of review | # articles | Search range | Language | Databases | Measures included | Summary of quality appraisal findings | Summary of review findings | PRISMA  score |
| --- | --- | --- | --- | --- | --- | --- | --- | --- | --- | --- |
| Desmedt et al | 2018 | To give an overview of empirical studies using self-reported instruments to assess patient safety culture in primary care and to synthesise psychometric properties of these instruments. | 14 | Until Nov 2016 | English, Dutch | Medline, Web of Science, Embase, | Anticipation & preparedness  -Staff safety climate surveys | Five studies used a self-reported questionnaire that was validated in a separate study  Internal consistency was assessed in all eight studies, with, respectively, two studies obtaining an excellent score and three studies obtaining a fair or poor score  Structural validity was assessed in six studies. One study obtained an excellent score. Two studies obtained a good score and three studies obtained a fair score.  COSMIN checklist used to evaluate the methodological quality of the instrument’s validation process.  Content validity was assessed in three studies, all obtaining a poor score.  Structural validity was assessed in six studies: excellent (n=1), good (n=2), fair (n=3).  The SCOPE-PC had the highest scores on the COSMIN scales | -A standard and widely validated survey is needed to increase generalisability and comparability.  -The SCOPE-PC survey is the most appropriate instrument to assess patient safety culture in primary care. However, further psychometric techniques are now essential to ensure that the instrument provides meaningful information regarding patient safety culture.  -There is a need to consider the triangulation of both qualitative and quantitative methods to attain a more in-depth assessment of culture. | Yes: 8 items |
| Authors | Year | Aim of review | # articles | Search range | Language | Databases | Measures included | Summary of quality appraisal findings | Summary of review findings | CASP |
| Madden et al | 2020 | To identify patient-report safety climate measures described in the literature, analyse the included items to consider their alignment with previously established safety climate domains, evaluate their validity and reliability, and make recommendations for best practice in using patient-report measures of SC in health care. | 44 (10 specific to primary care) | Until Nov 2019 | English | Medline, EMBASE, CINAHL, Academic Search Complete, PsycINFO | Anticipation & preparedness  -Patient safety climate surveys | A checklist of appraisal criteria was developed by the research team that addressed validity, reliability, and usability.  A score was awarded for each appraisal element deemed present, resulting in a possible total appraisal score from 0 to 9.  Variance in the validity, reliability, and usability of the measures was evident between 0% and 77.7% of appraisal criteria were fulfilled. Three measures (Patient Measure of Safety [PMOS], PMOS-10, and Child Hospital Consumer Assessment of Healthcare Providers and Systems [Child HCAHPS]) attained 77.7% of the criteria, and one measure achieved none of the criteria. | -Few measures reported satisfactory levels of validity, reliability, or usability measurement.  -The shorter variations of the original PMOS (PMOS-30 and PMOS-10) seem to be the most suitable valid, reliable, and acceptable measures of patient safety in secondary care. However, as yet, it is not yet possible to recommend a valid, reliable, and acceptable measure for use in primary care settings.  -Most measures were designed for use by patients in secondary care, with far fewer designed for measuring the attitudes of primary care patients.  -Given the value of using a mixed-methods approach when measuring patient safety, future research therefore may wish to further explore issues emerging from SC via interview  or through other qualitative engagement with patients. | Yes: 8 items |

| Authors | Year | Aim of review | # articles | Search range | Language | Databases | Measures included | Summary of quality appraisal findings | Summary of review findings | CASP |
| --- | --- | --- | --- | --- | --- | --- | --- | --- | --- | --- |
| Vasconcelos et al | 2018 | To conduct an investigation of the tools used to assess safety culture in primary care. | 18 | 1998- 2015 | English, Portuguese, Spanish, French | CINAHL, Science Direct, PubMed, BIREME lilacs, SciELO | Anticipation & preparedness  -Staff safety climate surveys | -Cronbach’s alpha ranged from 0.56-0.98 across tools.  -Confirmatory factor analysis conducted on 4/7 tools.  -Test retest reliability examined on one tool.  -Three tools looked at correlation scores. | -In addition to reliability, other measures of validity are needed to establish the credibility of an instrument. Research addressing other types of psychometric tests is needed.  -When choosing an instrument, a researcher must pay attention to measures that indicate if the chosen assessment tool is valid and the extent to which the instrument is adequate to the context in which it is supposed to be used.  -The domains of communication, management perception, and teamwork were present in all instruments. Future research on patient safety should incorporate these attributes. | Yes: 6 items  No: 2 items (research question not specific; insufficient quality assessment |

| Authors | Year | Aim of review | # articles | Search range | Language | Databases | Measures included | Summary of quality appraisal findings | Summary of review findings | CASP |
| --- | --- | --- | --- | --- | --- | --- | --- | --- | --- | --- |
| **Reporting systems only** | | | | | | | | | | |
| King et al | 2010 | To identify the state of the art in patient reporting systems used in research study, and review the healthcare setting populations, contact methods, verification, reporting methods, incentives, incident rates and terminology used for patient reports of adverse events. | 17  (5 primary care) | 1949-2008 | English | OVID Medline, PubMed | Past harm  -Patient reports of adverse events | None provided | -Further research is required to identify the optimal language, method of report solicitation, reporting tool and incentive for report completion.  -When designing a reporting tool it should be evaluated in the local setting to ensure appropriate terminology is used. International terminology standards should be adopted. Reports from patients should be actively solicited. | Yes: 6 items  No: 2 items (<3 databases searched; insufficient quality assessment |
| Ricci-Cabello et al | 2015 | To identify and characterise available patient reported instruments to measure patient safety in primary care | 28 | Up to July 2012 | No language limits  (English, Spanish, Dutch, French, German, Croatian, Italian) | Medline (Ovid), Embase, CINAHL, ISI Web of Knowledge, Health Management Information Consortium, LINNEAUS Euro-PC | Past harm  Patient reporting of adverse events | There was evidence for face validity for 10 instruments (43%), whereas evidence for construct validity was available only for 3 instruments. These instruments were also the only ones for which there was evidence of their reliability. | -Taxonomies for classifying errors and harm were not consistently used for developing the instruments.  -There was a lack of valid and reliable instruments to provide a comprehensive measurement of the safety of care provided in primary care practices.  -There is a lack of patient reported instruments for measuring patient safety that are comprehensive, psychometrically robust, and specific to primary care. | Yes: 7 items  No: 1 items (research question not specific) |

| Authors | Year | Aim of review | # articles | Search range | Language | Databases | Measures included | Summary of quality appraisal findings | Summary of review findings | CASP |
| --- | --- | --- | --- | --- | --- | --- | --- | --- | --- | --- |
| **Patient record review only** | | | | | | | | | | |
| Davis et al | 2018 | To understand the ability of trigger tools to detect preventable adverse events in the primary care outpatient setting/ | 15 | 1946-Feb 2017 | English | Ovid MEDLINE and Ovid MEDLINE In-Process and other non-indexed citations. | Past harm  Trigger tool patient chart review | The National Heart, Lug, and Blood Institute (NHLBI) Quality Assessment Tool and the Standards for Reporting Diagnostic accuracy studies (STARD) tool were used.  -Quality assessment showed a moderate risk of bias.  -Using NHLBI tool, 4 rated as “Good”, 6 rated as “Fair” and 5 rated as “Poor”.  -Using STARD tool, 4 rated as “Good”, 6 rated as “Fair” and 5 as “Poor”. Percent agreement between reviewers was 84% | -Accurate trigger tools remain elusive, primarily because of the high number of false positives detected with current tools.  -Inpatient care can use a single admission as representative of an entire episode, whereas outpatient care is a time-limited representation of a continuum of care, much of which is unobserved. However, identifying adverse events in the outpatient setting will require a combination of robust identification mechanisms such as trigger tools, scoring systems, chart reviews, and perhaps patient reporting.  -Outcome measures were heterogenous, precluding the ability to quantitatively compare the studies.  -Results support the notion that reliability of preventability assessment is lower than that of adverse events identification, although the interrater reliability for both varied widely. | Yes: 8 items |
| Authors | Year | Aim of review | # articles | Search range | Language | Databases | Measures included | Summary of quality appraisal findings | Summary of review findings | CASP |
| Madden et al | 2018 | To synthesize the literature describing the use of patient record review to measure and improve patient safety in primary care. | 15 | Until Feb 2017 | English | Medline, Embase, CINAHL, PsycInfo | Past harm  Patient record review | Quality was assessed using the Quality Assessment Tool for Studies with Diverse Designs (QATSDD).  The mean QATSDD score was 19.67 (SD 5.02; range 10–30) out of 48. Examining the QATSDD for each methodology:  -Trigger tool: 22.2  -Error definitions: 17.6  -Clinical judgement: 17  -Patient report: 20 | -A Patient Record Review (PRR) approach is a feasible and useful means of measuring, and potentially improving, safety in general practice. However, there is a need for future research to refine methodologies and ensure adequate training of practitioners to conduct PRR and to action the resulting data.  -Studies using trigger tool methodologies tended to detect higher incidences of PSIs, suggesting greater empirical support than other methods.  -There is a need to refine and standardise the methods used in PRR to improve consistency and validity and facilitate ease of comparison across studies.  -Considering the varying nature of PSIs detected according to method, there is strong rationale for combining more than one method of studying patient safety. | Yes: 8 items |

| Authors | Year | Aim of review | # articles | Search range | Language | Databases | Measures included | Summary of quality appraisal findings | Summary of review findings | CASP |
| --- | --- | --- | --- | --- | --- | --- | --- | --- | --- | --- |
| Tsang et al | 2012 | To determine the types of adverse events that are routinely recorded in primary care | 15 | Until Aug 2009 | English | ASSIA, Cochrane Library, EMBASE, HMIC, ISI Web of Science, Medline, PsycInfo, grey literature, websites | Past harm  Patient record review | -Evidence of quality assessment was not provided by three studies.  -Multi-reviewer assessments were conducted in approx. half of studies where data were extracted from medical records or incident reports (n = 6/11).  -All studies with calculations of inter-rater agreement reported good or excellent scores except in one study.  -Nine publications contained details of inclusion and/or exclusion criteria used and two- thirds of studies described the statistical methods that were applied. Recruitment methods included voluntary participation by self-selected general practices, sampling from single or multiple hospital sites and evaluations using population-based data collected nationally.  -The validity of adverse event measures was seldom assessed in the descriptive studies. | -Measurement of primary care adverse events was often based on secondary care data in conjunction with other clinical and non-clinical information. This use of multiple data sources will enhance the accuracy of measurements and compensate for weaknesses inherent to individual data types.  -Greater attention must now be placed on developing indicators and other measures that take advantage of the available IT resources to improve quality and safety. | Yes: 7 items  No: 1 item (insufficient quality assessment) |
