# Supplementary material for: A meta-review of methods of measuring and monitoring safety in primary care
Source: Int J Qual Health Care. 2021 Aug 18;33(3):mzab117. doi: 10.1093/intqhc/mzab117 (PMC8397923; doi:10.1093/intqhc/mzab117)
Supplement: mzab117_Supp [file mzab117_supp.zip › Supplimental material 1.docx]

**Supplemental Material 1.** Example search strategy for Medline (OVID)

*Search terms for Primary care*

1. Exp Primary Health Care/
2. Primary adj1 care.ti,ab.
3. Primary Health?Care.ti,ab.
4. Primary healthcare.ti,ab
5. Primary health care.ti,ab
6. Primary health-care.ti,ab
7. Exp General practice/
8. General adj1 Practi*.ti,ab.
9. Exp Family practice/
10. Family adj1 Practi*.ti,ab.
11. Exp ambulatory care/
12. Ambulatory adj1 care.ti,ab.
13. Exp community health services/
14. Community adj1 care.ti,ab.
15. GP*.ti,ab.
16. Family adj1 doctor*1.ti,ab.
17. Family adj1 physician*1.ti,ab.
18. 1 OR 2 OR 3 OR 4 OR 5 OR 6 OR 7 OR 8 OR 9 OR 10 OR 11 OR 12 OR 13 OR 14 OR 15 OR 16 OR 17

*Search terms for patient safety*

1. exp Patient Safety/
2. (patient*1 adj1 safe*2).ti,ab.
3. (safe*2 adj1 care).ti,ab.
4. exp Patient Harm/
5. (patient* adj1 harm*).ti,ab.
6. (preventable or avoidable or unnecessary) adj1 harm.ti,ab.
7. exp Iatrogenic Disease/
8. (iatrogenic adj1 disease).ti,ab.
9. (adverse adj1 event*1).ti,ab.
10. (sentinel adj1 event*1).ti,ab.
11. (safe* adj1 inciden*).ti,ab.
12. exp Medical Errors/
13. (health?care adj1 error*).ti,ab.
14. (medica* adj1 error*).ti,ab.
15. (human* adj1 error*).ti,ab.
16. (diagnos* adj1 error*).ti,ab.
17. (near-miss*2).ti,ab.
18. (near-failure*1).ti,ab.
19. (close-call*).ti,ab.
20. (incident* or voluntary) adj1 report*.ti,ab.
21. (safe* adj2 manag*).ti,ab.
22. (safe* adj2 culture*).ti,ab.
23. (safe* adj2 climate*).ti,ab.
24. (safe* adj2 attitude*).ti,ab.
25. (safe* adj2 behav*).ti,ab.
26. 19 OR 20 OR 21 OR 22 OR 23 OR 24 OR 25 OR 26 OR 27 OR 28 OR 29 OR 30 OR 31 OR 32 OR 33 OR 34 OR 35 OR 36 OR 37 OR 38 OR 39 OR 40 OR 41 OR 42 OR 43

*Search terms for methods*

1. survey*1.ti,ab.
2. questionnaire*1.ti,ab.
3. measur*.ti,ab.
4. monitor*.ti,ab
5. instrument*1.ti,ab.
6. eval*.ti,ab.
7. assess*.ti,ab.
8. examin*.ti,ab.
9. tool*1.ti,ab.
10. interview*.ti,ab.
11. checklist*1.ti,ab.
12. report*1.ti,ab.
13. 45 OR 46 OR 47 OR 48 OR 49 OR 50 OR 51 OR 52 OR 53 OR 54 OR 55 OR 56

*Search terms for systematic reviews*

1. “Review”/ or “Systematic Review”/
2. systematic* adj2 review).ti,ab
3. Meta?analysis.ti,ab.
4. (meta adj1 analy*).ti,ab.
5. metaanaly*.ti,ab.
6. Systemat*.ti,ab.
7. 58 OR 59 OR 60 OR 61 OR 62 OR 63
8. 18 AND 44 AND 57 AND 64

1680 results in Medline
